# The continuum of hepatitis C care for criminal justice involved adults in the DAA era: a retrospective cohort study demonstrating limited treatment uptake and inconsistent linkage to community-based care

**DOI:** 10.1186/s40352-017-0055-0

**Published:** 2017-10-30

**Authors:** Karli R. Hochstatter, Lauren J. Stockman, Ryan Holzmacher, James Greer, David W. Seal, Quinton A. Taylor, Emma K. Gill, Ryan P. Westergaard

**Affiliations:** 1 Department of Medicine, University of Wisconsin School of Medicine and Public Health, 1685 Highland Ave, Madison, WI 53705 USA; 2 Wisconsin Department of Health Services, Division of Public Health, AIDS/HIV Program, 1 W Wilson St, Madison, WI 53703 USA; 3 State of Wisconsin Department of Corrections, Bureau of Health Services, 3099 E Washington Ave, Madison, WI 53704 USA; 4 Tulane University School of Public Health and Tropical Medicine, 1440 Canal Street, New Orleans, LA 70112 USA

**Keywords:** Hepatitis C virus, Incarceration, Direct-acting antivirals, Co-morbidities, Linkage to care, Continuum of care

## Abstract

**Background:**

Incarcerated populations are disproportionately burdened by hepatitis C virus (HCV) infection. The introduction of highly-effective, direct-acting antiviral (DAA) treatment has potential to substantially reduce the burden of liver disease in this population, but accurate information about access to and utilization of this treatment is currently limited. The goals of this study were to characterize receipt of HCV care and treatment services for a cohort of HCV-infected adults identified in a state prison system, and to describe the complex health needs of this population.

**Methods:**

To estimate the proportion of patients who were treated for HCV while incarcerated, and the proportion linked to HCV care after release from prison, we used a deterministic matching algorithm to link administrative prison data, health care records, and a state public health surveillance database, which captures all positive HCV-related diagnostic test results through automatic laboratory reporting. Individuals not evaluated or treated for HCV while in prison were considered likely to have been linked to care in the community if the HCV surveillance system contained a record of a quantitative HCV RNA or genotype test within 6 months of their release date. Demographic and comorbidity data were manually extracted from the electronic health records for all patients referred for consideration of HCV treatment.

**Results:**

Between 2011 and 2015, 3126 individuals were known to be living with chronic HCV infection while incarcerated in the state prison system. Of these, 570 (18%) individuals were evaluated for HCV treatment while incarcerated and 328 (10%) initiated treatment with DAAs. Of the 2556 individuals not evaluated for treatment, 1605 (63%) were released from prison during the 5 year study period. Of these, 138 (9%) individuals engaged in HCV care in the community within 6 months. Data describing medical and psychiatric co-morbidities were available for the prison-based treatment cohort, which showed a high prevalence of major depression (39%), anxiety disorder (24%), alcohol misuse (52%), cocaine use (52%) and prior injection drug use (62%).

**Conclusion:**

Despite HCV treatment advances, linkage to care and treatment rates for criminal-justice involved adults remains low, particularly for those who must seek care in the community after release from prison. Treating criminal-justice involved individuals for HCV during incarceration provides an opportunity to improve linkage to care and treatment rates among this vulnerable population.

## Background

Chronic hepatitis C virus (HCV) infection is the leading cause of cirrhosis and liver cancer and the most common reason for liver transplantation in the United States. Left untreated, chronic HCV can result in serious health complications including liver damage, liver failure, liver cancer, and death. Approximately 19,000 people die annually as a result of a HCV-related liver disease (Centers for Disease Control and Prevention 2016a). Due to a high lifetime prevalence of injection drug use, incarcerated populations are disproportionately burdened by chronic HCV (Spaulding et al. 2006). Compared to a prevalence of about 1% in the general U.S. population (Denniston et al. 2014), adults residing in prisons experience a prevalence ranging from 10 to 41% (Varan et al. 2014). An estimated one-third of HCV-infected individuals in the U.S. pass through the correctional system, placing this system in a key position to control the HCV epidemic (Varan et al. 2014).

Incarcerated populations are also overburdened with psychiatric illnesses, chronic diseases, and substance use disorders. The Bureau of Justice Statistics estimated that approximately half of all prison inmates are affected by at least one mental health condition (James and Glaze 2006) and epidemiologic studies show that 15–24% of the U.S. prison population is affected by a severe mental illness (Teplin et al. 1996; Diamond et al. 2001; National Commission on Correctional Health Care 2002). Two large systematic reviews consistently demonstrated a wide range of drug and alcohol dependence estimates among prisoners, ranging from 10 to 60%. Sources of heterogeneity included gender, trends in drug use over time, and studies included in the reviews (Fazel et al. 2017). These illnesses serve as risk factors for unsuccessful engagement in medical care (Dixon et al. 2016; Kramer et al. 2012) and may play a role in the low HCV treatment uptake among those with such co-occurring disorders. One study of VA patients found that alcohol and drug use and depression were key reasons for not initiating HCV treatment (Kramer et al. 2012). Epidemiological research on the co-occurrence of these health issues among HCV-infected incarcerated populations is scare.

Since the introduction of the first HCV protease inhibitors in 2011, HCV treatment has evolved at a rapid pace. Direct-acting antiviral (DAA) drugs are now available which can cure HCV-infected individuals after 12 weeks of treatment with little to no side effects (American Association for the study of Liver Diseases and the Infectious Diseases Society of America 2017). DAAs, which have a cure rate of more than 90% and are typically very well-tolerated, offer new hope to reduce the burden of HCV in correctional institutions (He et al. 2016). HCV treatment in correctional settings has been shown to be both feasible and cost effective (Liu et al., 2014), and incarcerated patients have been shown to be as likely as non-incarcerated patients to successfully complete treatment and achieve a sustained virologic response (SVR) (Rice et al. 2012).

Despite HCV therapeutic advances, challenges remain for HCV screening and linkage to care. Investigations conducted around the US have found that a low proportion of individuals who test positive for HCV are linked to care and few receive treatment (Bourgi et al. 2016; Hochstatter et al. 2017; Viner et al. 2015). Two large population-level studies of HCV in the United States conducted by the CDC, the Chronic Hepatitis Cohort Study (CHeCS) and the National Health and Nutrition Examination Survey (NHANES), demonstrate that 32–38% of those who test positive for HCV antibodies receive follow-up HCV care and 7–11% are treated (Moorman et al. 2013; Spradling et al. 2012; Denniston et al. 2012). This gap in the care continuum is often exaggerated in vulnerable populations such as people who inject drugs and justice involved individuals who are more likely to experience social stigma, unstable housing, institutionalization, provider concerns regarding adherence, and other clinical and individual-level barriers (Harris and Rhodes 2013; Zeremski et al. 2013). Monitoring and developing strategies to optimize the HCV continuum of care remains an important public health priority.

Up-to-date and reliable information on the HCV continuum of care among correctional populations since the introduction of DAAs is limited. Routine and/or opt-out testing programs upon intake to prison, coupled with automatic laboratory reporting processes, provide the opportunity to collect information on the HCV epidemic among incarcerated populations. These processes ensure HCV-infected individuals cycling through correctional facilities are accounted for in public health surveillance systems, and thus provide useful tools for evaluating the HCV care continuum for individuals as they transition in and out of the criminal-justice system (Hochstatter et al. 2017). The overall goal of this study was to characterize the receipt of HCV care and treatment services for a cohort of HCV-infected adults identified in a single state prison system. By analyzing several, linked data systems, we specifically aimed to (1) determine the proportion of HCV-infected individuals who engaged in care with a HCV specialty provider while in prison; (2) describe these individuals who engaged in HCV care based on this evaluation; and (3) estimate the frequency with which unengaged individuals were linked to HCV care in the community after they were released.

## Methods

The study sample was comprised of adult individuals known to be living with chronic HCV infection while incarcerated in the Wisconsin Department of Corrections (WI DOC) between January 2011 and December 2015. Wisconsin is a medium-sized state in the Midwest region of the United States with an overall population of 5.7 million and an estimated HCV prevalence of approximately 1%. Using the available data sources described below, we conducted a retrospective cohort study to describe the continuum of care for HCV. Linkage of administrative data shared by WI DOC with clinical data from the electronic health records system of a large academic medical center and surveillance data from a state health department, we characterized HCV care engagement and treatment.

### Study setting

The WI DOC operates 36 adult institutions throughout the state and is responsible for approximately 22,918 individuals under custody at a given time. This population is 94% male, and among males, 53% identify as White, 43% identify as Black, 35% have a mental health condition, and 68% report having completed high school, have a High School Equivalency Diploma or a General Educational Development (GED), or have completed some post-secondary education (State of Wisconsin Department of Corrections 2017a). In 2016, 40.2% of females and 25.4% of males were admitted to prison for an active drug offence. Between 2000 and 2016, there was a significant rise in prison admissions for opioid and amphetamine drug offenses, while offenses related to cocaine and marijuana have declined or remained steady (State of Wisconsin Department of Corrections 2017b). Between 2007 and 2016, the percentage increase for females (19.7%) was approximately 2.5 times the percentage increase for males (7.8%). The prevalence of HCV in the WI DOC is estimated at 12.5% overall and is nearly two times higher in women facilities than men facilities (23.1% compared to 11.0%) (Stockman et al. 2016).

The WI DOC has two intake processing centers, one for women and one for men (aged ≥18). Providers offer HCV screening to newly incarcerated patients as part of the standard health evaluation according to risk- and age-based criteria. Risk-based criteria include having a history of injection drug use, a history of liver disease, elevated liver enzymes, human immunodeficiency virus (HIV), or being hepatitis B virus (HBV) core antibody positive. Following a system-wide seroprevalence survey in 2014, WI DOC officials added having a birth date between 1945 and 1965 to this list of criteria, which is estimated to capture 92% of HCV cases while screening 37% of the incoming population (Stockman et al. 2016).

The WI DOC’s HCV treatment criteria are consistent with guidelines established by the WI Medicaid Program. In all years of the study period, HCV treatment was prioritized for patients with stage F3 or F4 fibrosis, as indicated by liver biopsy or ultrasound-based elastography with a cut off value of 8.0. All DOC patients meeting these criteria are considered eligible for treatment if they are expected to remain incarcerated throughout the recommended duration of treatment. Patients with contraindications to treatment and those with less than stage F3 fibrosis are re-assessed yearly with laboratory testing and ultrasound elastogram. From 2011 to 2013, treatment consisted of combinations of Pegylated Interferon, Ribavirin and Telaprevir, depending on the patient’s genotype. The WI DOC was prescribing an average of 100 treatment courses per year and treatment drop-out rates were high due to the intolerable side effects of these medications. In 2013, when interferon-free, DAA-based regimens became available, the rate of treatment completion and sustained virologic response increased substantially, and treatment became available for patients with psychiatric conditions and heart disease that were previously considered contraindications to treatment.

### Data sources

**Fig. 1:**
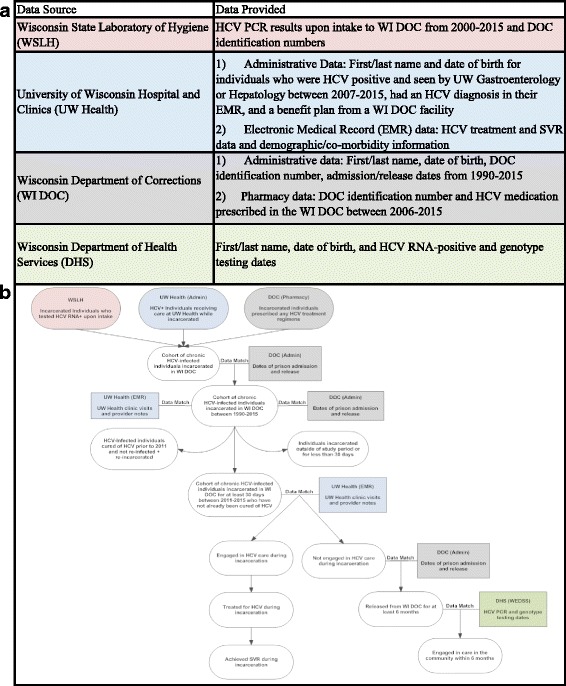
**a** Data sources and type of data provided from each source. **b** Process of database linkages for the identification of our study cohort and assessment of the HCV continuum of care

Figure 1a describes the four sources of data used for this study. Figure 1b displays the flow of database linkages for the identification of our study cohort and assessment of the HCV continuum of care.

### State laboratory of hygiene

Screening HCV antibody and confirmatory HCV ribonucleic acid (RNA) tests collected upon intake to DOC are conducted by the Wisconsin State Laboratory of Hygiene (WSLH), which has maintained a database of test results since 2000. We obtained a list of all DOC identification numbers and HCV RNA collection dates for those who tested positive upon intake between 2000 and 2015. The DOC number was then matched to a unique individual’s name and date of birth, and the resulting dataset was shared with University-based researchers.

### University-based hospital records

Information about liver disease staging and HCV treatment initiation was collected through electronic medical records (EMR) from the University of Wisconsin Hospital (UW Health), the academic medical center which has provided all HCV-related treatment for patients incarcerated through WI DOC since 2000. We cross-matched the list of HCV-infected patients from DOC facilities with a UW Health dataset containing names and dates of birth of all patients who attended a clinic visit in the Gastroenterology and Hepatology division between 2007 and 2015. To investigate whether any eligible patients were missing from the data provided by DOC, we also searched the UW Health EMR for patients attending hepatology clinic visits for whom WI DOC was listed as the insurance type or payer. Finally, we manually reviewed the charts of all patients to confirm eligibility for the analysis and exclude individuals who were cured of HCV prior to 2011.

### Department of corrections administrative and pharmacy data

The WI DOC pharmacy department provided a list of DOC identification numbers, first and last names, and date of birth for individuals prescribed medications used for the treatment of HCV between 2006 and 2015. After identifying all individuals incarcerated in a WI DOC facility with chronic HCV (HCV RNA) using these three databases (WSLH, UW Health EMRs, and WI DOC Pharmacy), we matched everyone to WI DOC administrative data for the identification of incarceration periods (Fig. 1b). The matching variable used DOC identification number. We included all individuals with chronic HCV (HCV RNA) infection who were incarcerated in one of the WI DOC prison facilities for at least 30 days between January 1, 2011 and December 31, 2015. Individuals were excluded if their EMR indicated they had already been cured of HCV and if they were incarcerated outside of the study period or for less than 30 days.

### Public health surveillance system

A subset of this cohort (described below) was matched to the Wisconsin Electronic Disease Surveillance System (WEDSS) using a deterministic record matching program developed by the Wisconsin Department of Health Services (DHS) HIV/AIDS Program staff using SAS® Version 9.3. The deterministic match used first name, last name, and date of birth variables to create an identifier to link the two datasets. Per Wisconsin Statute 252.05, any health care provider with evidence that a patient has HCV is required to report it to the appropriate local health department (Wisconsin State Legislature 2016). This information is then forwarded to the Wisconsin Department of Health Services (DHS) and stored in WEDSS. All positive HCV laboratory results are captured by WEDSS, allowing for the identification of HCV care in the community.

### HCV continuum of care

Two researchers jointly reviewed UW Health EMR for all individuals in the cohort to determine (1) which individuals attended a clinic appointment for evaluation of their HCV infection; (2) which completed a staging evaluation to characterize the extent of their liver disease; (3) which were prescribed antiviral therapy, and (4) which completed therapy and/or achieved SVR. SVR was defined as absence of viremia within 12–24 weeks after completion of antiviral therapy as indicated in the EMR by a UW Health laboratory report or a provider note. This flexible definition of SVR was used because of changes in practice during the study period, where assessment of viremia at 24 weeks was recommended during earlier years and 12 weeks was recommended in later years (Yoshida et al. 2015). EMR data was manually entered into a research database designed specifically for this study.

To understand linkage to care for those who do not receive HCV care during incarceration, we captured testing information after unengaged individuals released from prison. Using WEDSS data, we defined care engagement as evidence of any positive HCV RNA test or genotype test within 6 months of release from prison. For individuals with several incarceration periods, we used the first incarceration period after testing HCV RNA positive that lasted at least 30 days. Post-release engagement in care was not assessed for those re-incarcerated within 6 months of release.

In addition to HCV treatment and SVR data, we extracted demographic and co-morbidity data from the EMRs for each individual engaged in care at UW Health during incarceration. The primary source of this information was a detailed health questionnaire that is administered to patients through a standardized process and uploaded to each individual’s EMR. This questionnaire is primarily delivered by a Physician Assistant in the UW Health Gastroenterology and Hepatology Clinic and consists of questions about viral hepatitis risk factors, previous and ongoing substance abuse, and other health complications (Rice et al. 2012). We considered all diagnoses listed on this questionnaire in the present study. Chronic disease diagnoses included chronic obstructive pulmonary disease (COPD), chronic kidney disease (CKD), cirrhosis, cancer, diabetes, heart disease, HBV, HIV, and hypertension. Mental health diagnoses included anxiety or panic disorder, bipolar disorder, depression, and schizophrenia or other psychotic disorder not otherwise specified (NOS). Information on substance use disorders for this study was limited to the three categories listed on the questionnaire: injection drug use, alcohol dependence, and cocaine use disorder. These questionnaires were completed at each patient’s first appointment. Provider notes were also examined to collect information on diagnoses or risk factors that developed after the patient’s first encounter. Among those engaged in care at UW Health during incarceration, chi-square tests were conducted to determine whether there were any significant differences in demographic and co-morbidity variables between those who received HCV treatment and those who did not. Chi-square tests were performed using SAS 9.4 (Cary, NC) and descriptive statistics were gathered using Stata 15 software® (College Station Texas).

Encrypted data from WSLH, DOC, UW Health, and DHS, was shared with the research team at the UW Department of Medicine through a secure data-transfer system with appropriate Data Use Agreements in place. This research was approved by the Tulane University Human Research Protection Program Institutional Review Board, the University of Wisconsin Health Sciences Institutional Review Board, and WI DOC Research Review Committee.

## Results

### Study population

The WSLH database captured 3002 individuals with chronic HCV infection who underwent screening and confirmatory testing upon intake. An additional 162 individuals were identified by searching clinical databases at UW Health and pharmacy databases at WI DOC. UW Health EMR data revealed that 38 of these 3164 individuals had received interferon-based treatment and were cured of HCV prior to 2011, and were excluded from this analysis. The remaining 3126 were determined to have active HCV infection during the study period and were included in the cohort.

### Frequency of prison-based HCV treatment

**Fig. 2:**
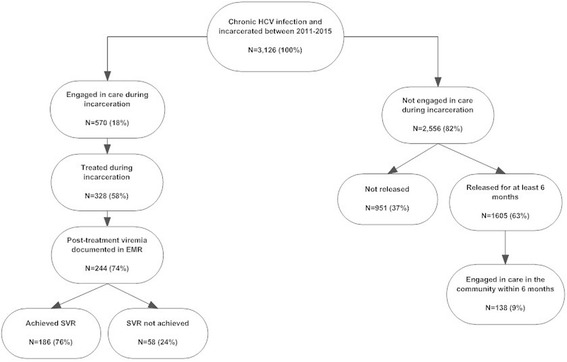
Individuals incarcerated in the WI DOC between 2011 and 2015 according to their stage of HCV care

**Fig. 3:**
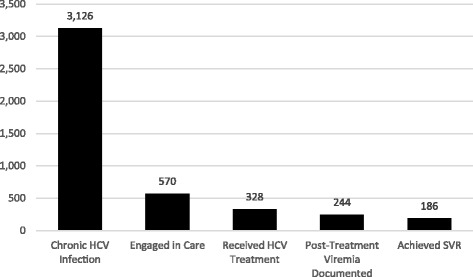
HCV care continuum for individuals in the WI DOC between 2011 and 2015 who engaged in HCV care during incarceration

Using the WI DOC prison population as of December 31, 2015 (22,918), the estimated prevalence of HCV among this population is 14%. Figure 2 illustrates the individuals included in this analysis according to their stage of HCV care. Of the 3126 HCV RNA positive individuals, 570 (18%) engaged in HCV care at UW Health while incarcerated. Based on review of the EMR for these 570 individuals, we determined 328 (58%) received HCV treatment. Of these, 244 had documentation of quantitative HCV RNA within 12 and 24 weeks after treatment completion in the UW Health EMR. Documentation of follow-up HCV RNA was missing for some patients whose clinical specimens were analyzed by an outside reference lab and not imported into the UW Health EMR. Of the 244 with available HCV RNA documentation, 186 (76%) had documented SVR and 58 (24%) had evidence of persistent viremia (Fig. 3). Investigation of the 58 who did not achieve SVR led to the discovery that 41 (71%) of these individual’s treatment regiments included pegylated interferon and ribavirin during early phases of the study period, which is known to have a lower cure rate and increased side effects compared to DAA based treatments (Manns et al. 2001). Among those with documented SVR, 117 (63%) were prescribed a DAA either alone or in addition or pegylated interferon and ribavirin.

### Frequency of linkage to community-based HCV treatment

Of the 3126 HCV RNA positive incarcerated individuals, 2556 (82%) were released from prison between 2011 and 2015 without having received evaluation for HCV treatment. Of the 1605 individuals who were released from prison and lived in the community for 6 months or more, we successfully matched 1594 (99%) to a record in the WEDSS database, indicating that future HCV-related laboratory test results would likely be captured. Evidence of a PCR or genotype test within 6 months of release were identified for 138 (9%) individuals, indicating they were likely engaged in medical care in the community.

UW Health medical records abstraction allowed us to describe the demographic characteristics and prevalence of co-morbidities among the 570 individuals engaged in care during incarceration (Table 1). Of these individuals, 91% were male, 70% where white, 24% were black, and 54% were born between 1945 and 1965, a birth-cohort with the largest share of chronic HCV infection (Centers for Disease Control and Prevention 2016b). There were no significant difference in any demographic variables between those who received HCV treatment and those who did not. Diagnostic procedures, including liver biopsy, CT scan, MRI, and Fibroscans, were reported in the EMRs for 225 individuals. A review of the results of these procedures revealed that 94 (42%) of these 225 individuals had advanced fibrosis or cirrhosis.

**Table 1:** Characteristics of cohort engaged in HCV care at UW Health during incarceration (2011–2015)

	Overall N (%)	HCV Treated N (%)	HCV Untreated N (%)	*P*-Value
Total	570 (100%)	328 (58%)	242 (42%)	
Male	518 (91%)	294 (90%)	224 (93%)	0.2302
Hispanic/Latino^a^	46 (9%)	22 (7%)	24 (10%)	0.2045
Race^b^				
White	378 (70%)	216 (70%)	162 (69%)	0.6467
Black	130 (24%)	75 (25%)	55 (24%)	0.7540
Other	28 (5%)	13 (4%)	15 (6%)	0.2693
1945–1965 birth cohort	308 (54%)	176 (54%)	132 (55%)	0.8337
Chronic conditions				
Cirrhosis	110 (19%)	65 (20%)	45 (19%)	0.7148
HIV coinfection	27 (5%)	7 (2%)	20 (8%)	0.0007*
HBV coinfection	7 (1%)	1 (0.3%)	6 (2%)	0.0198*
Hypertension	175 (31%)	100 (30%)	75 (31%)	0.8974
Heart Disease	25 (4%)	17 (5%)	8 (3%)	0.2794
Diabetes	88 (15%)	50 (15%)	38 (16%)	0.8810
CKD	14 (2%)	8 (2%)	6 (2%)	0.9755
COPD	20 (4%)	8 (2%)	12 (5%)	0.1061
Cancer	26 (5%)	14 (4%)	12 (5%)	0.6962
Psychiatric disorders				
Anxiety/Panic	138 (24%)	81 (25%)	57 (24%)	0.7532
Bipolar	26 (5%)	15 (5%)	11 (5%)	0.9875
Depression	220 (39%)	131 (40%)	89 (37%)	0.4434
Psychosis NOS	17 (3%)	8 (2%)	9 (4%)	0.3746
Substance use disorders				
Alcohol dependence	294 (52%)	176 (54%)	118 (49%)	0.2474
Cocaine use	319 (56%)	188 (57%)	131 (54%)	0.4490
Injection drug use	355 (62%)	217 (66%)	138 (57%)	0.0262*
Advanced Fibrosis/Cirrhosis^c^	97 (43%)	61 (63%)	36 (63%)	0.1138

*Statistically significant at α = 0.05

^a^Data on ethnicity was not recorded for 39 patients. Percentages are out of those whose ethnicity information was recorded (total *N* = 531)

^b^Designation of race was not recorded for 32 patients. Percentages are out of those whose race information was recorded (total *N* = 540)

^c^Of the 570, diagnostic procedure results were recorded for 225 (39%). Diagnostic procedures included Liver biopsy, CT scan, MRI, and Fibroscan. Advanced fibrosis/cirrhosis percentages are out of those whose diagnostic results were recorded (total *N* = 225). Advanced fibrosis/ cirrhosis was identified by a Metavir score of 3 or 4 using a liver biopsy or Fibroscan, or interpretation of advanced cirrhosis or fibrosis on a CT scan or MRI by the physician

The most prevalent chronic disease diagnoses were hypertension (31%), cirrhosis (19%), and diabetes (15%). Depression and anxiety were common psychiatric disorders, at 39% and 24%, respectively. Substance use disorders were also common, including alcohol dependence (52%), cocaine use (58%), and a history of injection drug use (62%). Those who received treatment were less likely to have HIV-coinfection (2% vs 8%) and HBV co-infection (0.3% vs 2%), however caution must be taken when interpreting these results as the low prevalence of HIV and HBV likely influenced statistical significance. Participants with a history of injection drug use were statistically significantly more likely to be treated; however, differences were minimal (66% vs 57%).

## Discussion

Despite remarkable advances in HCV treatment, chronic HCV infection remains a major public health problem. This study used EMR and automatic laboratory reporting data to evaluate HCV care engagement among incarcerated individuals during incarceration and after release from prison. The prevalence of HCV in the WI DOC estimated in this study (14%) is similar to that reported by Stockman et al. (2016) (12.5%). We found that 570 (18%) of the 3126 HCV-infected inmates engaged in HCV care during incarceration, 328 (10%) received treatment, and of the 244 whose treatment outcomes were documented in the EMR system 12–24 weeks after treatment initiation, 186 (76%) achieved SVR. This SVR rate is expected of a cohort treated both before and during the DAA era. A higher success rate would have likely been found if only studying those treated with a DAA, as indicated by the finding that the majority of individuals who did not achieve SVR had received pre-DAA medications.

Previous studies have demonstrated a high correlation between laboratory testing and medical care visits, providing validation for the use of HCV RNA test results to measure HCV care engagement (Rebeiro et al. 2013). Of the 1605 that released from prison without receiving HCV care and resided in the community for at least 6 months, we estimated that only 138 (9%) engaged in care in the community. True engagement in community HCV care is likely even lower than 9% because HCV viral loads may be have been ordered by any provider without the intention of monitoring and treating for HCV. Because automatic laboratory reporting of positive HCV tests was the only data source available to follow individuals after release, we were unable to determine how many individuals received treatment in the community. These results demonstrate that those who release from prison without receiving HCV care are very unlikely to engage in care in the community, a step in the HCV continuum of care rarely described in the current literature. This evidence, along with previous studies demonstrating feasibility and cost-effectiveness (Liu et al. 2014; Moorjani et al. 2015), provides further support for treating criminal justice involved individuals for HCV during incarceration. Not only does providing treatment during incarceration offer an opportunity for these individuals to access medical care, but also allows for directly observed therapy and controlled medication distribution in a structured environment, all methods for improved adherence.

As hypothesized, this study population experienced a high prevalence of chronic diseases, psychiatric disorders, and substance abuse. Previous studies have found that individuals with psychiatric and substance use disorders often fail to engage in medical care and complete treatments on their own (Dixon et al. 2016; Kramer et al. 2012). The high prevalence of these disorders among this incarcerated population may partially explain the low rates of care engagement. One limitation of this study is that comorbidity information was only available through one data source, UW Health EMRs, which only allowed us to assess the occurrence of these disorders among WI DOC patients engaged in care during incarceration. This lack of data prevents us from understanding whether the presence or absence of co-morbidities is associated with care engagement during incarceration. Future studies are needed to understand the causal link between these co-occurring disorders and HCV linkage to care.

Among those who were engaged in care at UW Health during incarceration, the prevalence of chronic conditions, mental health illnesses, and substance abuse disorders were similar between those who received HCV treatment and those who did not. The higher prevalence of HIV and HBV coinfection among those HCV-untreated raises concern as these diseases often cause worsening liver function. However, the low prevalence of these diseases among this study population warrants further research into whether treatment rates differ among those with different co-occurring disorders.

This study offers additional advantages over previously reported studies. First, using a variety of data sources to identify our study sample allowed for the construction of a complete cohort likely to capture all HCV infected individuals incarcerated across the state of Wisconsin. Because all WI DOC patients receive care at the same clinical practice, UW Health, completeness of the subset of individuals engaged in HCV care during incarceration can also be ensured. Moreover, DOC patients are often seen by the same few healthcare providers, which allowed for consistent data entry and ease of EMR data collection.

Using UW Health EMR data, we were able to exclude individuals who received treatment at UW Health and were cured of HCV prior to the study, which would include all WI DOC patients treated. Based on published literature demonstrating low HCV treatment uptake (Harris and Rhodes 2013), we expect the number of individuals cured in the community to be very small. A limitation of WEDSS is that mandatory reporting was only required for positive HCV tests at the time of this study. Automatic laboratory reporting of undetectable HCV viral load data to the state surveillance system would have allowed us to estimate how many individuals were treated and cured of HCV outside of UW Health while living in the community. Undetectable viral load data would also allow us to determine whether treatment was successful or not for those treated during incarceration whose post-treatment viral load results were missing from EMR data (*n* = 84). Despite these limitation of WEDSS, this study found that 99% of individuals who released from prison without receiving HCV care during incarceration were identified in WEDSS. This provides evidence of an efficient surveillance system and support for using mandatory laboratory reporting to study the HCV care continuum.

WI DOC relies on Wisconsin Medicaid criteria for the development of policies surrounding treatment for HCV. As Wisconsin Medicaid standards have recently expanded to treat more individuals with HCV, an increasing number of individuals within the WI DOC have gained access to HCV treatment. Expanding Medicaid access is also important for this population as this is the largest source of health insurance coverage for criminal-justice involved individuals living in the community. The Wisconsin Medicaid program has worked with WI DOC to increase Medicaid enrollment prior to release from prison. Currently over 70% of individuals releasing from the WI DOC have Medicaid coverage within 30 days of release from prison. Expanding Medicaid access and unrestricted reimbursement for DAAs is a necessary and promising strategy to improve linkage to care and treatment for this population. More research will be needed to examine the impact these policy changes will have on the HCV epidemic.

## Conclusions

Despite HCV treatment advances, linkage to care and treatment rates for HCV-infected, criminal justice involved adults remains low, particularly for those who must seek care in the community after release from prison. More research is needed to understand barriers former inmates face getting linked to care in the community and the role co-morbidities play in determining linkage to care and treatment uptake. Treating criminal-justice involved individuals for HCV during incarceration provides an opportunity to improve linkage to care and treatment rates among this vulnerable population, a key unmet goal necessary for HCV elimination. Unless improvements are made in linkage to HCV care and treatment uptake, advances in HCV therapy will continue to have a limited impact on the burden of HCV-related diseases in the population.

## Abbreviations

EMR: Electronic medical record
HCV: Hepatitis C virus
RNA: Ribonucleic acid
WEDSS: Wisconsin electronic disease surveillance system
WI DOC: Wisconsin department of corrections
WSLH: Wisconsin State laboratory of hygiene
